# Measuring close proximity interactions in summer camps during the COVID-19 pandemic

**DOI:** 10.1140/epjds/s13688-022-00316-y

**Published:** 2022-01-31

**Authors:** Elia Leoni, Giulia Cencetti, Gabriele Santin, Timofei Istomin, Davide Molteni, Gian Pietro Picco, Elisabetta Farella, Bruno Lepri, Amy L. Murphy

**Affiliations:** 1 DIGIS, Fondazione Bruno Kessler, Via Sommarive 18, 38123 Trento, Italy; 2 DEI, University of Bologna, Viale del Risorgimento 2, 40136 Bologna, Italy; 3 DISI, University of Trento, Via Sommarive 9, 38123 Trento, Italy

**Keywords:** Close proximity interactions, Contagion risk levels, Social bubble strategy, Wearable devices

## Abstract

Policy makers have implemented multiple non-pharmaceutical strategies to mitigate the COVID-19 worldwide crisis. Interventions had the aim of reducing close proximity interactions, which drive the spread of the disease. A deeper knowledge of human physical interactions has revealed necessary, especially in all settings involving children, whose education and gathering activities should be preserved. Despite their relevance, almost no data are available on close proximity contacts among children in schools or other educational settings during the pandemic.

Contact data are usually gathered via Bluetooth, which nonetheless offers a low temporal and spatial resolution. Recently, ultra-wideband (UWB) radios emerged as a more accurate alternative that nonetheless exhibits a significantly higher energy consumption, limiting in-field studies. In this paper, we leverage a novel approach, embodied by the Janus system that combines these radios by exploiting their complementary benefits. The very accurate proximity data gathered in-field by Janus, once augmented with several metadata, unlocks unprecedented levels of information, enabling the development of novel multi-level risk analyses.

By means of this technology, we have collected real contact data of children and educators in three summer camps during summer 2020 in the province of Trento, Italy. The wide variety of performed daily activities induced multiple individual behaviors, allowing a rich investigation of social environments from the contagion risk perspective. We consider risk based on duration and proximity of contacts and classify interactions according to different risk levels. We can then evaluate the summer camps’ organization, observe the effect of partition in small groups, or social bubbles, and identify the organized activities that mitigate the riskier behaviors.

Overall, we offer an insight into the educator-child and child-child social interactions during the pandemic, thus providing a valuable tool for schools, summer camps, and policy makers to (re)structure educational activities safely.

## Introduction

Close proximity interactions (CPIs) drive the spread of any disease that is transmitted predominantly by respiratory droplets and saliva, such as influenza, common colds, and severe acute respiratory syndromes (i.e., Severe Acute Respiratory Syndrome (SARS), Middle East Respiratory Syndrome (MERS), Coronavirus Disease 2019 (COVID-19)) [1–6]. An improved characterization of CPIs should thus lead to a better understanding of the spread dynamics and possibly inform public health experts and policy makers to design more effective interventions [7].

For this reason, some research efforts have used wearable devices and Radio Frequency Identification (RFID) or infrared (IR) sensors to measure and analyze high-resolution proximity interactions in different settings such as schools [4, 8], workplaces [9, 10], hospitals [11–15], households [16], and conferences [9, 17, 18].

During the COVID-19 pandemic, social contacts and in particular CPIs were significantly modified [19–22] by several non-pharmaceutical interventions such as physical distancing measures (i.e., 1 m or more), mobility restrictions, closings of schools, universities, and selected businesses (e.g., restaurants, bars, coffee shops, gyms), promotion of teleworking, cancellations or limits on the size of events (e.g., sports events, weddings, funerals), limits on the number of people in small family, educational and social gatherings (i.e., social bubbles), etc. [23–25].

However, despite their relevance, almost no data are available on how CPIs occur among children in contexts such as schools or summer camps during the COVID-19 pandemic, thus making it difficult to evaluate and model the effects of physical distancing measures, small group strategies, preferences for outdoor activities, masks, etc., on CPIs, as well as identifying the situations and activities during school and summer camp days where the risk of transmission is elevated.

The collection of reliable data in these environments (e.g., schools, summer camps) is itself a nontrivial task. During the pandemic, several local and national governments have launched smartphone digital contact tracing (DCT) apps based on the Bluetooth Low Energy (BLE) technology [26] and the GAEN (Google and Apple Exposure Notification) interface [27], and several studies have shown the effectiveness of Bluetooth-based DCT using real-world contact patterns [28, 29] and in pilot and country-wide studies conducted in Switzerland, the United Kingdom (the Isle of Wight and the whole country), and Spain (Gomera island) [30–33].

In addition to the challenge that most children do not carry personal smartphones, this technology has at least two shortcomings for capturing CPIs in schools and summer camps: (i) *low temporal resolution* (e.g., GAEN detects neighbors every 4 minutes [27]), and (ii) *low spatial resolution*, which directly descends from limitations of BLE and leads to significant estimation errors [34]. The first issue can be tackled by the use of an alternative to GAEN, while the second can be addressed by changing the technology used for estimating distances, e.g., to ultra-wideband (UWB), which brings the spatial error down from meters to decimeters [35].

In this paper, we address these issues via a novel approach, embodied in the Janus system [36], combining a custom, efficient device discovery mechanism based on BLE with the ability to accurately measure pairwise distances via UWB. In our experiments, we configured Janus to acquire distance measurements every 30 s and installed it on a wearable device that children can easily carry. We have collected real-world CPIs with Janus at three summer camps in the province of Trento (Italy). These camps offer interesting settings because of the rich variety of daily activities that induce different CPIs among children and between children and the summer camps’ educators. Moreover, the summer camps took place during the summer of 2020, in the middle of the pandemic and just after the local easing of lockdown measures. As such, it is possible to investigate the effect of the guidelines and regulations enforcing physical distancing, mask-wearing, outdoor activities, and the formation of small groups (i.e., social bubbles).

The accurate and fine-grained contact data uniquely enabled by Janus, complemented by the metadata about summer camps, results in the rich data set that is the basis of our multi-level analysis. First, we explore the definition of *close contact* as the aggregation of multiple raw measurements captured by the sensors and discuss the modeling choices implied by this operation. After this aggregation phase, the resulting contacts are enriched with metadata. For example, social bubbles [37, 38] were enforced as a contagion containment measure, and thus we assign to each contact the groups of the two involved individuals. Further, each contact is associated with the activity being performed during the contact time.

By considering the metadata in the analysis along with the raw contact data, we offer novel insights into both educator-child and child-child social interactions during the pandemic. In particular, we study the distribution of the level of contagion risk among individuals depending on the proximity and duration of their contacts, finding that a vast majority of CPIs are classified as low risk. Moreover, we aggregate the contacts as intra-group (i.e., within the social bubble) and inter-group (i.e., between different bubbles), and observe changes in the distribution of contact risk levels in the two cases, offering evidence of the effectiveness of the social bubble strategy [37, 38]. Finally, a thorough analysis of the different activities provides insights into their inherent risks of contagion, which can be further interpreted in view of the features of the activity itself (indoor or outdoor, static or dynamic, etc.).

The results of our analyses provide information immediately actionable by school and summer camp managers and teachers, policy makers, and public health experts.

## Related work

In this section we review key works related to our paper from two distinct areas: (i) the development of technologies for detecting proximity contacts, and (ii) the usage of close proximity data for modeling the spread of infectious diseases.

### Detecting proximity contacts

Several technologies have been explored for detecting proximity, e.g., including infrared [39], ultrasound [40], and IEEE 802.15.4 [41]. However, one of the earliest and most popular systems for measuring and tracking close proximity interactions, proposed in the context of the SocioPatterns project, was based on the pairwise exchange of active RFID signals among badge-like devices [9]. In this case, distance measurements were estimated every 20 s based on the received signal strength of packets transmitted at multiple power levels, and an estimation of face-to-face context was provided based on the receipt of a very low power signal.

The approach of estimating pairwise distances based on signal attenuation is common in radio-based systems [41], notably including COVID-19 contact tracing apps [42] relying on the low-cost and low-energy BLE chips pervasive in smartphones. A similar approach is also exploited by many commercial BLE-based tags offering contact tracing functionality in scenarios where a smartphone is not available or practical. Unfortunately, in both cases the low accuracy of distance estimation with BLE, whose approach based on signal strength is significantly affected by environmental factors [35, 43], remains a major issue [34].

This motivated interest in UWB, recently exploited by several contact tracing and proximity tags. However, the significantly higher distance estimation accuracy of UWB is counterbalanced by its higher energy requirements, about an order of magnitude higher than BLE. For example, the Bump contact tracing UWB tag [44] has been employed in large-scale events such as the London marathon; however, the higher UWB energy consumption limits the tag lifetime to 12 hours. Further, the mechanics of UWB distance estimation, involving a pairwise packet exchange [45], require coordination among nearby nodes to avoid packet collisions, whose negative impact on reliability can be significant. For instance, the scheme in [46] reports a 65% success in scenarios with 10 neighbors.

In contrast, our work utilizes the Janus system that applies both the UWB and BLE radios in concert. By using the energy-efficient BLE radio for constant discovery operations as well as for coordination, Janus ensures that the expensive UWB radio is activated only when strictly needed to acquire a distance estimate, and in a coordinated way that significantly mitigates the impact of collisions. Further details about Janus are found in Sect. 3.1 and in [36].

### Modeling the spread of infectious diseases from proximity data

The analysis of proximity contact data includes multiple works that focus on modeling the spread of infectious diseases. For instance, Salathé et al. [4] have used sensors with a proximity resolution up to 3 meters in a high school to obtain a dataset in which they have simulated the spread of an influenza-like disease. Doing this, they have found results in agreement with absentee data during the influenza season.

Another line of studies has exploited data collected in different environments within the SocioPatterns project. In particular, the estimation of face-to-face interactions was used to correct theoretical epidemiological infectiousness parameters and thus to obtain a better risk estimation for generic spreading processes [12, 47–50], or to identify specific individual roles in workplaces, hospitals, schools that would be more responsible for the spread of a disease [16].

Other face-to-face interaction data have been collected by Duval et al. [14] to understand how hospital-acquired infections spread and to possibly design control strategies. Similarly, Obadia et al. [51] collected CPIs and data about a staphylococcus transmission in a hospital, finding that collected CPIs were able to correctly reproduce transmissions and thus demonstrating the importance of this tool to trace disease spread.

Additional studies have also focused on contact tracing strategies, such as the work by Farrahi et al. [52] and more recently, for COVID-19, the ones of Cencetti et al. [28] and Barrat et al. [29].

Finally, a few in-field experiences with Janus are reported in [36], also in the context of COVID-19. Nevertheless, these are meant to illustrate the possible uses of the system, have significantly shorter duration, and do not consider the interplay of contact data and the activity metadata.

Instead, our current study describes the collection of real-world daily educator-child and child-child close proximity interactions at three summer camps during the COVID-19 pandemic. This unique dataset allows us to characterize contagion risks based on duration and proximity of contacts and classify interactions according to different risk levels. We can then investigate the effect of the guidelines and regulations enforcing physical distancing, observe the effect of partition in small groups (i.e., social bubbles), and identify the summer camp activities that mitigate the riskier behaviors in terms of contagion.

## Materials and methods

Here, we concisely describe the salient aspects of the Janus system used in our in-field studies, offer details about the summer camps where they were performed and the mechanics of data acquisition, and state the definition of close proximity contact used throughout the paper.

### Janus: a system for measuring close proximity interactions

Janus [36] relies on a dual-radio architecture to provide an accurate and energy-efficient system for proximity detection. We split proximity detection into two primary functionalities: identifying the other devices nearby and measuring the distances between them.

The first, device discovery, must be performed continuously as people (and the devices they carry) move freely in an unconstrained space. Fundamentally, Janus detects that two devices are near each other when they are able to communicate. For this continuous operation, we exploit the lower power BLE radio and build atop the continuous Bluetooth Low Energy neighbor discovery protocol, BLEnd [53]. BLEnd defines the optimal schedules for the BLE advertisement and scan periods to minimize consumption while meeting a service level agreement defined by the maximum allowed latency to discovery, the required probability for discovery, and the maximum number of devices expected to be in range. In our in-field studies, we configured the BLEnd component of Janus to guarantee the discovery of a neighbor within 30 s at least 95% of the times, provided no more than 20 devices are in range.

Once a nearby device is detected, Janus exploits the payload of the BLE advertisements continuously sent by BLEnd to coordinate, at no additional communication cost, the accurate ranging between devices performed by the UWB radio. Janus relies on single-sided two-way ranging (SS-TWR), part of the IEEE 802.15.4 standard [45]. This scheme requires a 2-packet exchange between an initiator and a responder; the transmission and reception of these packets are timestamped and made available at the initiator, which can compute the time of flight and therefore the distance between devices. In Janus, each device periodically schedules a ranging window during which it is available to respond to ranging requests; the aforementioned coordination exploiting BLE advertisements informs each neighbor of its unique offset into this window, ensuring that ranging requests among multiple neighbors do not collide. Additional details about Janus are available in [36].

**Figure 1:**
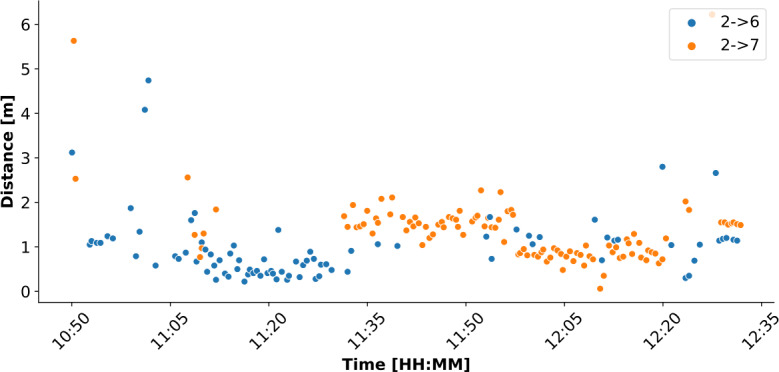
Raw data obtained from a Janus device. Timestamped distance measurements collected over 105 min by the Janus device of user 2 with respect to the devices of users 6 and 7

Figure 1 provides an example of the distance data directly obtained from Janus devices, which in our case are the MDEK1001 development kits by Decawave (now Qorvo), equipped with a BLE radio and the popular DW1000 UWB transceiver. The chart shows a snapshot of 105 minutes for device 2 with respect to two other devices, 6 and 7. Each dot indicates the distance measurement between device 2 and either device, color-coded as blue for device 6 and orange for device 7. According to our configuration, samples are taken every 30 s. Even without additional data processing, it can be easily seen that device 2 (and therefore the person carrying it) was very close (within 1 m) to device 6 for approximately 15 min, starting just after 11:00. We also note that the data is quite *clean*; the variations of the measurements across time are consistent. This is due to the accuracy of UWB, which enables our subsequent analysis.

### Data acquisition

The data used in our analyses are the results of a study conducted from August to September 2020 in three different summer camps, summarized in Table 1, in Trentino, Italy. The study design was approved by the Agency for Family, Birth, and Youth Policies (Agenzia Provinciale per la Famiglia, la Natalità, e le Politiche Giovanili) of the Autonomous Province of Trento,^1^ the provincial government body responsible for the organization of the summer camp programs, and by the two social cooperatives directly responsible for camp management and activities. In preparation for the study, parents and educators were provided with detailed information about the purpose of the study, the data treatment and privacy enforcement strategies, the devices the children and educators would be using, and the measurements they provide. Following Italian regulations, all parents and educators signed an informed consent form. Special attention was given to privacy and data protection: no personal information was associated with the identifier of the corresponding Janus device. We did note the group (i.e., social bubble) the individual belonged to and, in some cases, the identity of devices carried by others for whom physical distancing rules were waived (e.g., among siblings and between children with special needs and the educators assigned to assist them).

**Table 1:** Description of the three summer camps investigated in our study

ID	Short Description	Ages	Children	Educators	Groups
am-pri	Morning camp with a large indoor space, nearby a public park.	6-11	21	5	3
day-pri	All day camp in an alpine region with only outdoor space.	6-11	13	5	2
day-int	All day camp in an alpine region with additional indoor space.	11-14	9	2	1

The first summer camp, am-pri, operated for half days (mornings) with 21 primary school-age children and 5 adult educators, all of whom agreed to participate in the study. The children were divided into 3 groups, each with one or two educators. Each activity during the day was restricted to a single group at a time to maintain separation and leverage the concept of social bubbles [37, 38].

The second and third camps were organized the same week by the same cooperative, but took place at different locations; therefore, we treat them separately. Both were all-day camps from 8:00 to 16:30. day-pri applied the social bubble with two groups of primary school children. The third camp, day-int, involved 9 intermediate school children with two educators. The overall study participation rate in these two camps was 94%.

The summer camps engaged the children in different educational and playing activities, as summarized in Table 2. For each activity, we indicate the approximate duration in minutes for each camp.

**Table 2:** Daily activities at the summer camps, each with a brief description, the location and the duration in minutes for each summer camp that offered the activity

Activity	Description	Location	am-pri	day-pri	day-int
Woods	Playing in a wooded area	outdoor	90 min		
Soccer	Playing in a soccer field	outdoor	90 min		
Board games	Playing tabletop games	indoor	90 min		
Newspaper	Pairs work at computers	indoor	90 min		
Theater	Singing and acting	indoor	90 min		
Snack	Short food break	indoor	15 min		
Team games	Organized group games	indoor	90 min	120 min	120 min
Crafts	Arts and craft	indoor	90 min		180 min
Hiking	Group walk	outdoor			240 min
Round table	Greetings, planning, etc	indoor			180 min
Day closing	Free play pre pick-up	outdoor		30 min	60 min
Outdoor lunch	Eating	outdoor			60 min
Indoor lunch	Eating	indoor		60 min	
Free play	No organized activities	indoor			60 min
Free play	No organized activities	outdoor		60 min	

#### Device setup and experimental setting

**Figure 2:**
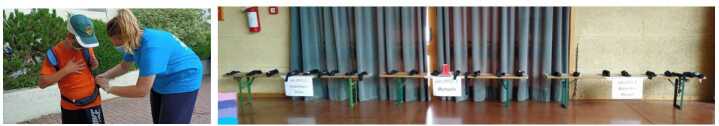
Janus device management at the am-pri camp. Left: An educator fitting the waist bag containing the device on a child, on the first camp day. Right: Devices in waist bags sitting on a storage bench overnight; the inhibitor device is inside the red bag in the center

To make carrying the device comfortable for the children, we inserted it inside a waterproof waist bag, as shown on the left of Fig. 2. We received positive feedback from the educators, who said that the children immediately forgot they were wearing the device. As mentioned, the Janus device is configured to sample distances every 30 s when devices are in proximity. Measurements greater than 10 m are discarded to save memory on the device and because these large distances are not considered relevant for the transmission of Severe Acute Respiratory Syndrome Coronavirus 2 (SARS-CoV-2) [54, 55].

After programming the devices and inserting new batteries, the waist bags were delivered to camp organizers at the beginning of each week. The educators were responsible for handing out the bags to the same children each morning and collecting them at the end of the day. At the end of the week, the devices were collected and the data offloaded via Universal Serial Bus (USB).

As the devices do not have an on/off switch, to avoid the collection of meaningless data at night, when devices were stored on a bench (Fig. 2), we implemented an *inhibitor* device. This special device was turned on at the end of the day by connecting it to a USB power bank. When the regular devices detected the BLE advertisement of the inhibitor, they went to sleep for 5 min. Upon restarting, if the inhibitor was detected again, they returned to sleep; otherwise, they started functioning normally, ranging with all neighboring devices. Each morning, the inhibitor device was detached from its power supply. This inhibition mechanism saved battery as well as memory and, most important, required no technical skills from the educators; even using the USB power bank was much easier than removing the battery from all Janus devices, which was the only other alternative available.

### Definition of close proximity contacts

After downloading the measurements from all devices, we pre-processed them as detailed in Appendix A. This processing removed spurious measurements, e.g., those recorded between the morning activation of the devices and the start time of the activities. We then aggregated these raw samples into *contacts* characterized by two device IDs, the timestamp marking the beginning of the contact, the contact duration, and a distance, as described next.

To identify a contact, we focus on a pair of IDs, collecting all measurements captured by either device, and sorting them in time. This sequence is then processed sequentially to divide the time into multiple, meaningful contacts. Intuitively, a contact should contain measurements that are all *temporally and spatially close* to one another, which we define via time and distance thresholds.

We begin with the temporal dimension, splitting the sequence into sub-sequences whenever a gap of $\tau _{\mathrm{time}}=90$ s exists between two consecutive measurements. This step accounts for interruptions in the interaction between the pair of devices, e.g., when they move away from one another.

Second, we check each of the distances inside each sub-sequence, ensuring that a single contact contains only measurements with similar distances, and ensuring that the single distance attribute assigned to a contact has a reasonable spatial variation. Therefore, we sequentially process the measurements of a sub-sequence in temporal order, and retain them in a single sub-sequence as long as all the measured distances are within $\tau _{\mathrm{space}}=2$ m from each other; a new sub-sequence is started upon the first measurement outside this range.

In this way, we obtain a set of sub-sequences, each containing measurements without large temporal gaps and with similar distances. After discarding sub-sequences with fewer than $\tau _{\mathrm{len}}=2$ measurements, we aggregate each cluster into a contact. Each contact is tagged with the timestamp of the first measurement in the sub-sequence, a duration given by the time span of the measurements in it, and a distance given by the median value of the measurements. Using the median (i.e., the central value of the distribution) yields a more robust value compared to the mean, which is more sensitive to extreme values and outliers.

**Figure 3:**
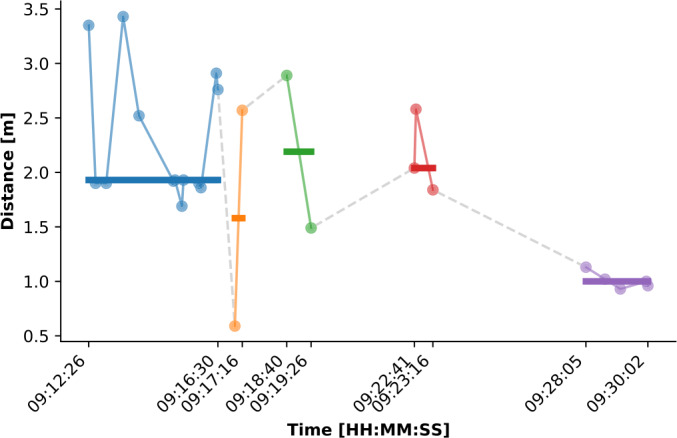
Measurement splitting and contact aggregation process. The figure shows the measurements collected in the first 20 min of August 8th, 2020, between node 26 and 27 at the am-pri camp. The measurements (light colors) are colored according to the division into contact characterized by $\tau _{\mathrm{time}} =90$ s and $\tau _{\mathrm{space}} =2$ m. Each contact is depicted as a horizontal bar from its beginning to its end, where the height of the bar represents the median distance

An example of this splitting and aggregation process is shown in Fig. 3, which depicts a sequence of measurements in a 20 min period grouped into sub-sequences (identified by colors) and aggregated into contacts (identified by the horizontal lines). The different splitting strategies can be observed. For example, the orange and green sequences are separated due to the gap of more than $\tau _{\mathrm{time}}$ between them. On the other hand, the blue and orange sequences are separated because the first measurement in the orange cluster is outside the range of $\tau _{\mathrm{space}}$ with respect to the previous measurements.

The resulting contacts model the high-level notion of CPI that we use in our analyses in the next sections, and enables the general contagion risk assessment of the different environments. Further, we also associate to each contact the groups of the involved IDs and an activity when both IDs are in the same group.

Some of the contacts can be removed a posteriori to account for risk-modelling choices. For instance, we discard contacts between siblings (who were not required to respect physical distancing rules) or between children with special needs and their support teacher. Additionally, in day-pri and day-int, the two activities “welcoming activity” and “swimming pool” have been discarded because the devices had not all been distributed and were piled up in the same place, resulting in many spurious measurements.

The resulting numbers of contacts for each summer camp setting are reported in Table 3. For each data set, we also report the number and percentage of contacts where both users belong to the same group, and thus to which we are able to assign an activity.

**Table 3:** Description of the contacts resulting from the aggregation procedure. For each camp, we report the total number of contacts, the average number of the measurements for each contact, the number of groups and activities in the camp, and the number and percentage of the contacts that are uniquely associated with an activity. For day-pri and day-int, we report both the number of activities, and the number of activities considered for the analysis (in parenthesis)

ID	Num. Contacts	Average Measurements per Contact	Num. Groups	Num. Activities	Activity-tagged contacts
am-pri	7259	5.80	3	8	6833 (94.13 %)
day-pri	7561	8.48	2	5 (4)	6774 (89.59 %)
day-int	3485	16.40	1	9 (7)	3485 (100.00 %)

## Results

Leveraging the previous definition of contacts and additional metadata, we can now delve into the analysis of the complex daily CPI patterns within the summer camps.

### Identification of contagion risk levels

To build a general model for risk analysis, we define four different categories of contagion risk for contacts based on proximity and duration. We then classify all contacts into these categories.

In a meta-analysis and systematic review of observational studies on Severe Acute Respiratory Syndrome Coronavirus (SARS-CoV), Middle East Respiratory Syndrome-related Coronavirus (MERS-CoV), and SARS-CoV-2 person-to-person transmission [55], a physical distancing of less than 1 m was reported to result in a significantly higher transmission risk than distances higher than 1 m (12.8% vs. 2.6%), thus supporting a minimum physical distance of 1 m, as in the rule enforced in schools and summer camps in Italy. However, as pointed out by Jones et al. [54], physical distancing rules would be more appropriate and effective if they offer graded levels of risk. Similarly, although contact tracing guidelines in several countries, various digital tracing contact apps, and some studies [56] assume that the duration of exposure to a person with COVID-19 influences the transmission risk (e.g., defining a threshold of 15 min beyond which transmission risk increases), a precise quantification of the duration of exposure is still missing [54].

Following these considerations, we define the risk categorization summarized in Table 4. The first category is associated with a *high risk* of contagion and includes all contacts with duration above 15 min and distance less than 1 m. The second category, *medium-high risk*, includes all contacts with duration above 10 min and distance below 2 m that are not included in the high-risk category. The third category, *medium-low risk*, includes contacts with duration above 5 min and distance below 4 m not included in the previous categories. The fourth category contains all remaining contacts, therefore associated to a *low risk* level.

**Table 4:** Risk levels of contagion defined on the basis of duration of exposure and physical distance

	Duration	Distance
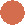 High risk	≥ 15 min	≤ 1 m
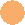 Medium high risk	≥ 10 min	≤ 2 m
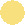 Medium low risk	≥ 5 min	≤ 4 m
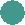 Low risk	< 5 min	> 4 m

Notably, this granularity in discriminating risk levels is enabled by the fine-grained spatio-temporal resolution offered by Janus. The high accuracy of UWB ranging, in contrast to the coarse, Received Signal Strength Indicator (RSSI) based distance estimation [43] with errors on the order of meters, enables spatial discrimination at the granularity of a meter. Similarly, our configuration of Janus captures distances every 30 s, while the popular GAEN interface collects a single sample in each 4 minute window.

It is worth noting that while our data is rich in terms of accuracy, the Janus platform does not capture whether interactions are face-to-face. However, while face-to-face interactions provide a good approximation of conversations and are useful for social interaction analysis [9, 57], when studying SARS-CoV-2 transmission this aspect is less critical. Indeed, several researchers are highlighting that SARS-CoV-2 can spread among people occupying the same space, whether or not they are facing one another [58, 59].

Further, while our definition of risk level is context-agnostic, based only on proximity and duration in line with the national and international policy recommendations [60, 61], our analysis in the following sections is context-aware as it takes into account metadata that notably includes whether or not the contacts occurred indoor or outdoor. This two-step strategy allows for an in-depth risk assessment and effective definition of the risk levels without requiring possibly intrusive and privacy-critical contextual information.

### Contagion risk analysis

**Figure 4:**
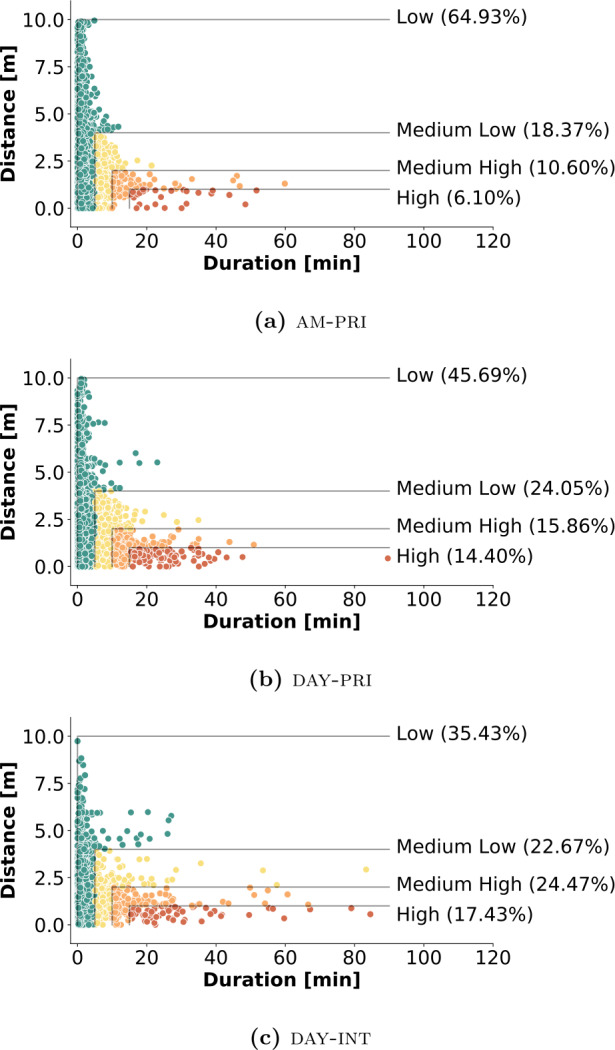
Summer camp contacts and contagion risk. The figure reports, for each summer camp, the corresponding contacts classified according to their risk of contagion as a function of the duration of exposure and proximity, following the risk categories in Table 4. The values in parentheses denote the percentage of time spent in a contact with the corresponding risk category

Figure 4 shows a scatter plot for each summer camp dataset, reporting the recorded contacts as a function of duration and proximity. Each dot represents a contact, as defined in Sect. 3.3, with colors describing the associated risk according to the color code in Table 4. The percentages reported inside the figures, and associated with the different risk levels, represent the percentage of time spent by the population in the corresponding risk category. Interestingly, we see that, even if different summer camps imply different levels of risk, there is a non-negligible percentage of contacts at high risk of contagion in all summer camps.

**Figure 5:**
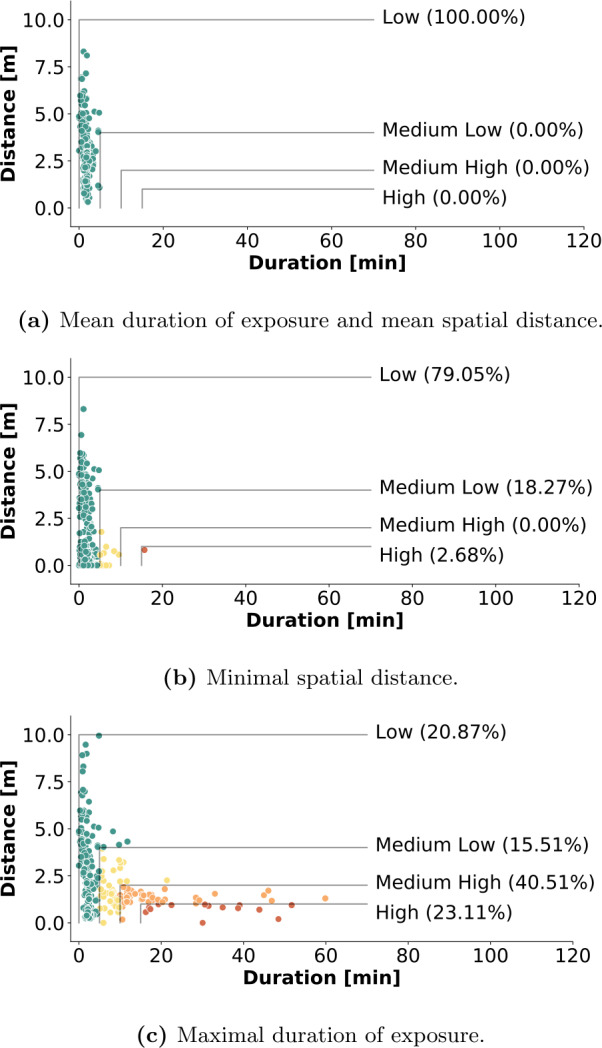
Unique contacts and risk levels. Contacts from am-pri, aggregated into a single point (one per device pair) according to different criteria

In the representation in Fig. 4, each dot represents a single contact between two individuals, but it ignores information about the corresponding IDs. Therefore, it is possible that the analyzed population has heterogeneous behaviors, e.g., with only a few participants involved in more risky close proximity interactions and the majority of individuals interacting safely, or vice-versa. To understand how the risk is distributed among the summer camp population we consider three additional views, shown in Fig. 5, where we examine the behavior for pairs of individuals. We report only the case of am-pri, since the other camps yielded analogous results. First, in Fig. 5(a), we compute for each pair the average distance and duration across all the contacts, resulting in a single dot per pair. We observe that each pair interacts, on average, in low-risk social interactions. A similar result is observed in Fig. 5(b), where we select the single contact per pair with the smallest proximity distance. Finally, Fig. 5(c) shows the single contact per pair with the longest duration. Here, we see that ∼23% of the pairs of individuals are involved in very high-risk interactions. From this, we conclude that the risk of contagion is distributed quite homogeneously among the different pairs of individuals, except for some for which the longest interactions are also the most dangerous ones.

These graphical representations give a first, general idea of the contact risk levels and offer an understanding of how the risk is distributed among the individuals. We note that these analyses depend on our definition of contact and, particularly, on the thresholds defined in Sect. 3.3.

In addition, the proposed contact definition allows us to perform two types of meta-analysis based on the risk levels related to: (i) group dynamics (e.g., CPIs among group members, among members of different groups, educator-child interactions, child-child interactions), and (ii) the type of educational and recreational activities planned during the summer camp.

**Figure 6:**
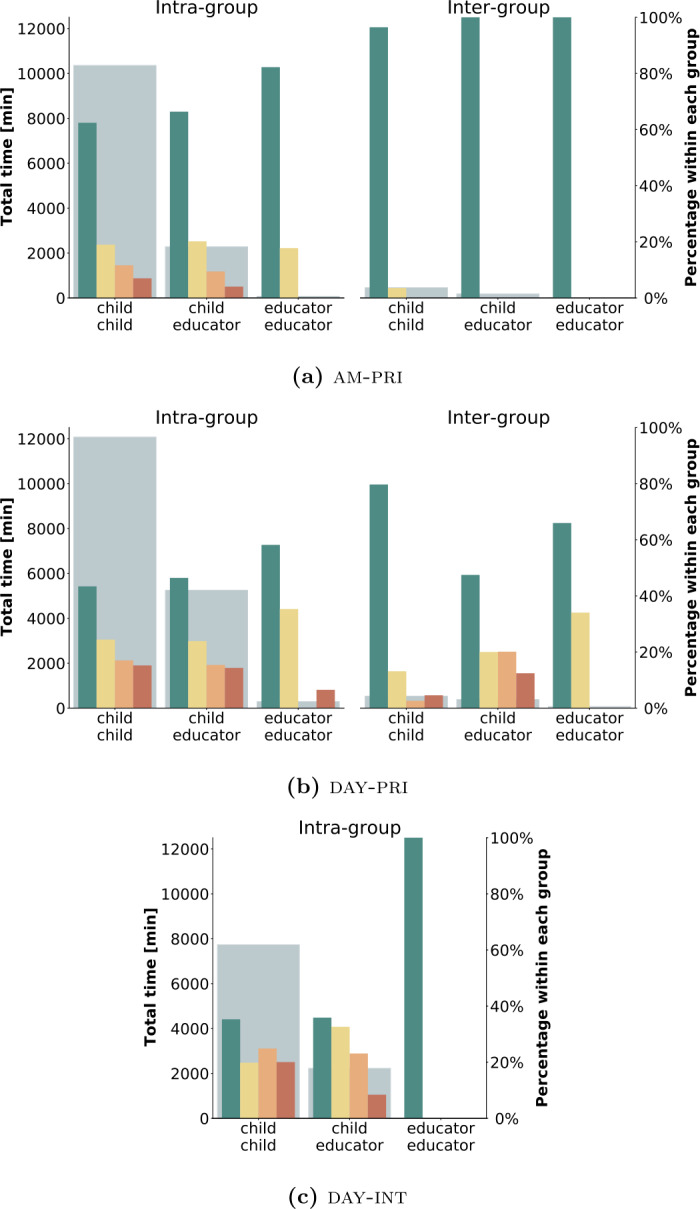
Social bubble policy and roles. Distribution of risk levels by group and type of interaction for each summer camp. The color bars, which refer to the right-hand scale, report the percentage of time of contact within each risk level. The grey background bars, which refer to the left-hand scale, report the total time of contact for each of the six categories

As described in Sect. 3.2, each summer camp setting organized participants in small groups and in specific roles (educator, child). Groups are intended to keep participants separated into disjoint bubbles [37, 38] so that any contagion event would remain localized. On the other hand, roles reflect the internal organization of the summer camps, where both children and educators were present. The results are graphically reported in Fig. 6, where the colored bars show the relative percentages of contacts for each risk level that can be attributed to child-child, educator-child, and educator-educator interactions, respectively. Moreover, these can be divided into interactions involving two people belonging to the same group (“intra-group”) and those bridging two different groups (“inter-group”). Instead, the large grey bars in the background report the total percentages of contacts for each specific type of interaction, independently on the associated risk. To facilitate the quantitative comparison of the results, Table 5 reports, for each summer camp, the number and the total duration of the contacts in the six groups.

**Table 5:** Summary of the number and duration of the contacts in the three camps according to the social bubble strategy. For each camp am-pri, day-pri, and day-int, we report for the different bubbles the total time of contact and the number of contacts organized by the role of the participants

		Intra-group			Inter-group		
		Child child	Child educator	Educator educator	Child child	Child educator	Educator educator
am-pri	Time [min]	10,362.40	2285.28	77.82	462.12	181.77	11.75
	Number	5484	1297	52	295	121	10
day-pri	Time [min]	12,075.02	5250.60	290.32	538.95	383.93	72.33
	Number	4388	2064	145	341	195	49
day-int	Time [min]	7732.58	2229.58	4.22	–	–	–
	Number	2004	613	2	–	–	–

**Figure 7:**
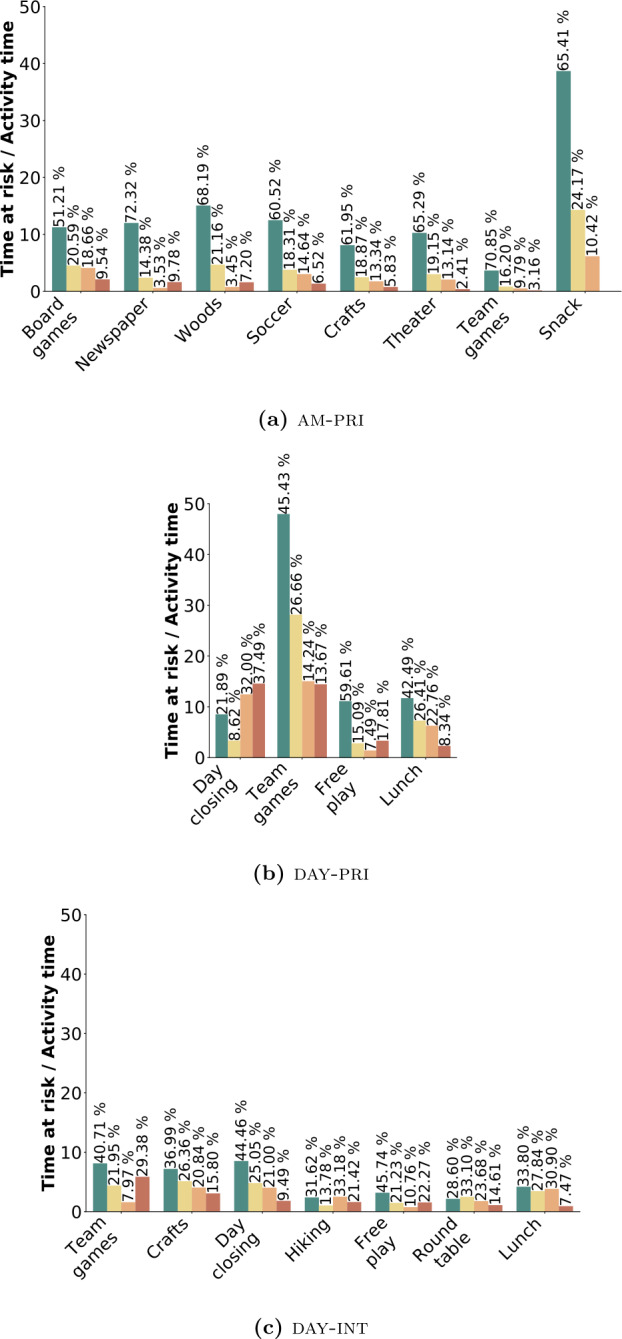
Activities and risk levels. The figure shows the distribution of the risk levels by activity, sorted according to a decreasing percentage of high-risk contacts for am-pri (Fig. 7(a)), day-pri age range 6–11 (Fig. 7(b)) and day-int 11–14 (Fig. 7(c)). The percentages show the fraction of contact time within each risk level, for each activity

When a contact occurs between two members of the same group, we assign to it the activity being performed at that moment by that group. In this way, we add another layer of analysis that allows us to study the relationship between the activity type, the number, and the contagion risk level of the contacts. The results are shown in Fig. 7, where we report four bars for each activity, representing the four risk levels. The height of the bars represents the sum of the duration of all contacts during each activity divided by the total duration of the activity. Hence, each bar reports the risk per unit time of each activity. This normalization allows comparison across the different activities, independent of their duration. The percentages show the fraction of contact time within each risk level, for each activity.

## Discussion

We already observed that in all summer camps there is a non-negligible percentage of contacts at high risk of contagion and that this is in general not due to some specific individuals or couples of individuals but the risk is quite homogeneously distributed among all the participants (Figs. 4–5). We now discuss more in detail the results and their implications.

### Social bubbles and roles

To analyze the effectiveness of the social bubble policies, we look at Fig. 6, which reports the percentages of contacts taking place inter- and intra- groups and between children and children, educator and educator, and educator and children for the three summer camps. Note that in day-int there was only a single group. We observe, as expected, that intra-group contacts are more numerous, but they are also interpreted as less risky since they are foreseen and permitted within the social bubble policies. On the other hand, inter-group contacts happen across different groups and are generally more risky; however, their limited number is a good indication of the effectiveness of the application of the social bubble policies. The collected data thus confirm that in case of an epidemic spreading in these settings, most of the possible contagions would likely be restricted to a single group, and transmission to other groups would be avoided or limited. Focusing on the interactions within each group, we observe that the highest percentages of contacts with high or medium-high risk of contagion involve children (i.e., children-children or educator-children CPIs), while the educators tend to have low-risk interactions among them.

### Activity type

For summer camp am-pri shown in Fig. 7(a), it is evident that the activity involving the highest number of interactions per unit time is “snack”; however, it is also the only activity where none of the CPIs was at high risk. This is actually by design as the activity duration is less than 15 min (Table 2), which is the minimum duration required to mark a contact as high risk (Table 4). We observe a similar finding in the other two data sets, day-pri and day-int (Figs. 7(b) and 7(c)), where “lunch” is the activity with the fewest risky contacts. This is probably because, during meal times, the children were not wearing their face masks; thus, the educators were paying more attention to the compliance to physical distancing rules. Moreover, the children were seated during lunch, so there was a reduced probability of accidental CPIs.

Other low-risk activities in am-pri were “crafts”, “theater” and “team games”, all meticulously organized activities where the educators established precise rules for physical distancing to avoid CPIs. The risk rises instead with “soccer” and “woods”, where no precise rules were established, and the children were free to move in a large space. Moreover, these activities took place outdoor, and there is evidence for a reduced transmission risk during outdoor activities as compared to indoor ones [62–65].

The riskiest activities, still with a limited total duration of high-risk close proximity contacts, are represented by “newspaper” and “board games”, two indoor activities with specific constraints: the first consisted of collaborating in pairs in front of a computer, working on the summer camp’s newspaper, and the second one consisted of playing board games around a table. Since the activities required being close to each other watching the same screen or table, the physical distance clearly could not be very large. However, it is worth highlighting that children wore face masks during the activities, thus reducing the transmission risks [66–68].

Moving to day-pri, a different summer camp with a different organization (Fig. 7(b)), we observe a high number of contacts during the activity “team games”, even if most of these contacts are at low risk of contagion. Interestingly, in this summer camp the organized games imply many more contacts per unit time with respect to “free play”. However, the activity with the highest percentage of high-risk CPIs is “day closing”, which was the final part of the day, when children were waiting for pick up and entertained themselves by playing table tennis or table football, in rather unstructured way.

An additional and final scenario can be observed in day-int, showing different typical behaviors, possibly due to a higher age range of the participants, namely 11–14 years old, and different adherence to physical distancing rules. Figure 7(c) shows a general lowering of the time spent interacting with each other and, at the same time, a higher percentage of high-risk CPIs. Differently from am-pri but similarly to day-pri, we observe that the activities with the highest risk are exactly the most organized ones: “team games” and “craft”, followed by the ones where children were more free to move around: “day closing”, “hiking”, and “free play”. The activities that provide less high-risk CPIs are instead “round table” and “lunch”, where participants were sitting to talk or eat, all together but keeping a well-defined physical distance from one another.

All together, this analysis of the activities shows the different ways in which different settings have been addressed. In particular, it seems that the combination of mask-wearing in the close-interaction static activities and a precise organization of the dynamic activities results into an overall effective strategy to contain the risk.

### Lessons learned and actionable policies

Our analysis clearly outlines the effectiveness of the social bubble policy and the multi-faceted nature of the risk implied by the different summer camp activities, which in turn highlights the importance of performing a fine-grained analysis of these activities. These analyses are made possible by a novel tool capturing distances and exposure duration during CPIs, and would not be reproducible with competing technologies, characterized by a lower spatial resolution.

We also envision the process of informing policy makers and schools through a number of actionable policy recommendations, especially when designing non-pharmaceutical interventions and contact tracing activities. Namely, special attention should be paid to a strict design and enforcement of a social bubble policy, which is a low cost action that requires almost no limitations to the behavior of the children. Moreover, via contact tracing it is possible to clearly identify which summer camp activities, given their risk levels, should be limited or require special attention. In our analysis, for instance, some should preferably require the presence of stable pairs (e.g., “newspaper”) and others should be moved outdoors (e.g., “board games”). This process should be planned as a continuous feedback loop, with periodic reassessments of the risk levels and a consequent redesign of the activities, enabled and informed by our accurate and non-invasive approach to contact tracing.

### Limitations

As with any experimental data collection, we acknowledge the limitations of our study. First, the gathered data sets are limited in time by the duration of the summer camps (one week, and half or whole days only) and by the number of participants (61 individuals in total). While the high temporal and spatial resolution enabled by Janus allow interesting analyses, the sample size and length limits make it impractical to simulate an epidemic spreading model based on this population. Further, all the summer camps were located in the Trentino area, and do not necessarily directly translate to other cities, regions, or countries, perhaps with different distancing rules.

Finally, a comparison to similar studies in the summer camp setting is not possible, as none are available in the literature. Moreover, we do not have hard ground truth to compare against; this would have required either cameras or manual annotations, which would have greatly interfered with the children privacy and the camps’ activities. Nevertheless, the results and findings we outlined have been shared with the educators, who confirmed them based on their knowledge and recollection of the activity organization, and the observed general behavior of the children and educators.

Despite these limitations, we reassert that the data collected by the Janus devices is, to the best of our knowledge, the only example of physical distance data for child interactions with high spatio-temporal resolution collected during the COVID-19 pandemic.

## Conclusion

Tracking and measuring CPIs in a real setting is a challenging task that, however, plays a crucial role in understanding the dynamics of social interactions during the pandemic and their effect on the spread of the disease.

This work shows that the Janus system is well-suited to provide high temporal and spatial resolution data to capture CPIs in complex settings like summer camps. Similar observations would have been impossible to obtain with either BLE or UWB alone.

In particular, we have analyzed three summer camps’ daily activities and social interactions in the Autonomous Province of Trento (Italy). The captured CPIs allowed us to derive several key insights into the duration and proximity patterns characterizing the child-child and the educator-child interactions.

Specifically, we verified the effectiveness of the social bubble strategy, which is easy to implement in the summer camp setting and offers an effective mechanism to balance control of the epidemic against light restrictions on the children during educational and recreational experiences.

Moreover, we analyzed the risk levels of a series of activities performed during the summer camps. We obtained key information into their safety in terms of number of contacts, duration of the contacts, and level of contagion risk. When combined with other metadata such as the location (indoor vs. outdoor) and the possibility to adopt personal protective equipment (i.e., face masks), this information can be exploited towards actionable policies to design safer environments for interactions among children in the summer camp setting but also at schools.

## Figures and Tables

**Figure 8:**
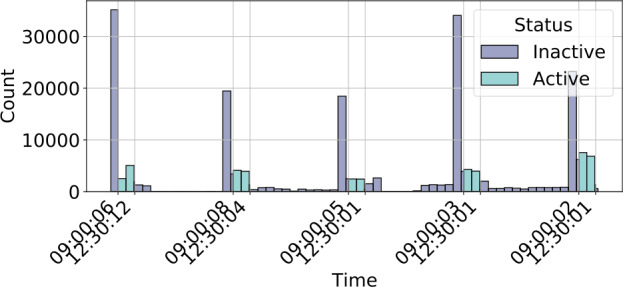
Filtering of the spurious measures. Distribution of the measurements over the entire sampling period, either with *Active* or *Inactive* status for am-pri

## Data Availability

The datasets generated and analysed during the current study are available from the authors on reasonable request. Please contact Bruno Lepri (lepri@fbk.eu) or Amy L. Murphy (murphy@fbk.eu).

## Appendix A: Pre-processing of the data

Prior to analysis, the data collected during each summer camp were cleaned of spurious samples recorded by the devices. We describe the process here and report a summary of the collected data for each setting.

The Janus devices do not have an on/off switch, and as a result, are active 24 hours per day, not only when the summer camps are in session. Although we used the inhibitor device to limit the measurements taken after the daily close of the summer camp, some additional measurements are still stored.

For example, if the BLE signal to the inhibitor was weak, the devices may have been briefly activated. Additionally, the inhibitor node was often disabled several minutes before children arrival and devices distribution, resulting in measurements among the devices still on the storage bench. Finally, some children were absent for entire days or arrived late while their device was still taking measurements.

Identifying all these cases was a largely manual effort based on information from the educators about absences and observations in the data itself. For example, when a sequence of constant distance measurements is seen at the beginning of the day, it is likely that the devices are still in storage, as children are rarely so still. The data cleaning step filters all these spurious measures. Table 6 shows for each summer camp the data collection time frame, the number of unique participants that have been involved, the number of overall measures, and the number of measures after the filtering step.

**Table 6:** Statistics of the raw data sets, including the number of measures before and after the pre-processing step

ID	Initial day	Final day	Unique users	Raw measures	Filtered measures
am-pri	2020-08-17	2020-08-21	24	222,222	48,739
day-pri + day-int	2020-08-24	2020-08-30	25	213,219	146,576

Figure 8 shows the distribution of the entire measurement set for am-pri. The time intervals during which the activities took place (*Active*) are separated from the time between the activities (*Inactive*). The peaks of data close to the morning camp start time correspond to the phase when the inhibitor node is off, but the devices have not yet been distributed to the children. In this case, all devices are immobile, near one another on a bench (Fig. 2) and thus save many distance measurements.

## Footnotes

1 https://www.trentinofamiglia.it/
